# Optimizing oncolytic reovirus therapy in hepatocellular carcinoma: A single dose enhances immune modulation and tumor delivery

**DOI:** 10.1016/j.omton.2026.201305

**Published:** 2026-08-07

**Authors:** Emma J. West, Karen J. Scott, Adel Samson

**Affiliations:** 1 Leeds Institute of Medical Research at St. James’s, University of Leeds, Leeds, UK; 2 Leeds Teaching Hospitals NHS Trust, Leeds, UK

## Oncolytic viruses as an effective immunomodulatory agent in cancer

Oncolytic viruses (OVs) have a multi-modal capability to cause death of cancer cells, while sparing normal cells; their primary mechanism of action being immune-mediated killing of tumor cells. Viral infection and subsequent lysis of tumor cells results in the release of cellular components, which activates the peripheral immune system. Upon activation, immune cells release inflammatory factors, such as interferons (IFNs), which induce a proinflammatory cytokine/chemokine milieu to drive both a rapid (innate) and prolonged (adaptive) antigen-specific response against malignant T cells, including migration of activated cells to the site of inflammation, i.e., the tumor site.^1^^,^^2^

OVs have been used in a multitude of clinical studies, both as a single dose and repeated doses, over a singular or several cycles, with three viruses having been approved for the treatment of cancer.^3^ Despite extensive investigations in humans, it is not clear what dosing schedule is optimal for both delivery of virus to the tumor site and initiation of anti-tumor immunity. Furthermore, the optimum route of delivery also remains inconclusive; intratumoural (*i.t*.) administration is achievable for many accessible tumors but is not possible for inaccessible tumors, therefore a systemic (intravenous; *i.v.*) approach is needed. Despite numerous studies via both routes of delivery, there is no published literature indicating the superiority of repeat dosing over a single dose.^4^

## Challenges in exploiting OVs in chronically damaged organs

While tumor cell lysis is associated with enhanced immune cell infiltration into the inflamed tumor site, the potential for associated immune cell influx into surrounding “normal” tissue exists. Some tumor-bearing organs have pre-existing damage due to chronic inflammation, with reduced capacity to cope with further inflammatory insults, including immune-mediated injury. Hepatocellular carcinoma (HCC) patients predominantly have underlying liver cirrhosis and lower hepatic reserves, resulting from chronic liver inflammation and damage sustained over a number of years. While many OVs have been tested in HCC, with varied results to date,^5^ a delicate balance between anti-HCC immunity and off-target immune-mediated damage to the cirrhotic background liver is paramount to therapeutic success. As such, rationalizing the delivery schedule of OVs to reduce unwanted background liver damage, while maximizing clinical benefit, is crucial in this patient cohort.

## A single dose of reovirus is as effective as repeated doses in inducing peripheral immune activation in patients

In a recent study published in *International Journal of Cancer*,^6^ our group investigated the peripheral immune response in patients from two clinical trials, where one group of patients received a single *i.v.* dose of pelareorep (reovirus)^7^ and the other group received repeated daily *i.v.* doses over a single cycle of five days.^8^ Each individual dose contained 1 × 10^10^ 50% tissue culture infectious dose (TCID_50_) of reovirus; in these clinical trials (and all our following *in vitro*/*in vivo* work), a single dose of reovirus was equal to each individual dose where repeated virus treatments were administered. We showed that a single dose was equally effective, if not superior, to repeated doses in activation of patients’ immune systems; specifically, in peripheral blood mononuclear cell (PBMC) subsets, including T cells, NK cells, monocytes, and B cells. In parallel, elevations in many inflammatory cytokines, including IFNs, were detected in patient plasma. Many of these solutes are known to be involved in the stimulation of T and NK cells in the induction of anti-tumor immunity. Conversely, a significant reduction in Th2 inhibitory cytokines, IL-4 and IL-5, was observed, indicating that the immune balance was predominantly toward inflammatory rather than inhibitory—interestingly, this was seen to a more significant level in those patients who only received a single dose of virus. Furthermore, key chemokines involved in immune cell chemotaxis, e.g., interferon gamma-induced protein 10 (IP-10), monokine induced by gamma interferon (MIG) and macrophage inflammatory protein 1-β (MIP-1β), were also induced post-reovirus infusion, with a single dose again being equivalent to repeat doses. We also demonstrate post-reovirus lymphopenia in both patient cohorts, as expected following systemic viral therapy,^9^ suggesting migration of lymphocytes out of peripheral circulation. Interestingly, a subcutaneous HCC model demonstrated effective delivery of reovirus to tumor in both cohorts but that repeated doses did not amplify the amount of virus that was detected in the tumor, indicating a single dose is sufficient. Notably, following repeated doses we observed a greater influx of T cells into both tumor and, more importantly, background liver, where this has the unwanted potential to result in immune-mediated damage.

Utilizing HCC patient PBMCs, we also show immune cell activation via elevation of CD69 (an early activation marker) and PD-L1 (a post-activation exhaustion marker), with repeated doses having no additional impact. Using our *in vivo* model, we show that following a single dose of reovirus, an increase in both CD69 and PD-L1 was evident in T cells, whereas PD-1 remained unchanged.

Collectively, these data indicate that a single reovirus dose is as effective as repeated doses in both delivery to tumor and immune activation, potentially resulting in an inflammatory immune environment with direct lymphocyte migration to the tumor site. The observation that repeated doses did not amplify any of these responses over a single dose indicates immune cell refractoriness^10^ following the initial stimulation.

## OV therapy in the context of HCC

To investigate the impact of oncolytic virotherapy on damaged background liver in the context of HCC, we utilized a C57/BL/6 model where we induced chronic steatohepatitis via a high-fat diet (HFD) or fibrosis via administration of a toxin (CCl4). Once liver damage was established, we injected the mice with single or repeat doses of reovirus. Equivalent increases in CD69 and PD-L1 expression on hepatic-resident T cells were observed across both groups, indicating no further enhancement of T cell activation with repeated doses. Liver function was significantly compromised; both single and repeated doses of reovirus induced elevated levels in aspartate aminotransferase (AST) and alanine transaminase in CCL4-treated mice. Moreover, in HFD mice, repeated doses increased AST levels to 2× the level induced by a single dose and 5× that of the healthy range, indicating additional damage to the liver, over that observed with a single dose.

To investigate the optimum route of virus delivery to target tumor immune activation, while avoiding potential background liver damage, we compared *i.v.* and *i.t.* injection. Although *i.t.* delivery induced a marginally greater increase in CD69 expression compared to *i.v.* delivery, no significant differences in PD-1 or PD-L1 were observed. However, due to changes in PD-1 and PD-L1 expression following virus infusion, we went on to investigate reovirus in combination with antibodies targeting these checkpoint molecules to enhance immune activation. As expected, we observed no impact of either single or repeated viral doses as a monotherapy on tumor growth or survival. Consistent with this, reovirus treatment of both HCC patient primary tumor cells and HCC cell lines also had very little effect on cell death *in vitro*. In combination with reovirus *in vivo*, anti-PD-1 did not impact the growth of tumors nor survival of the mice; however, in contrast, combination with anti-PD-L1 significantly delayed tumor growth and prolonged survival. More importantly, repeated doses had no additional therapeutic effect over a single dose using this combination.

## Conclusion

**Figure 1:**
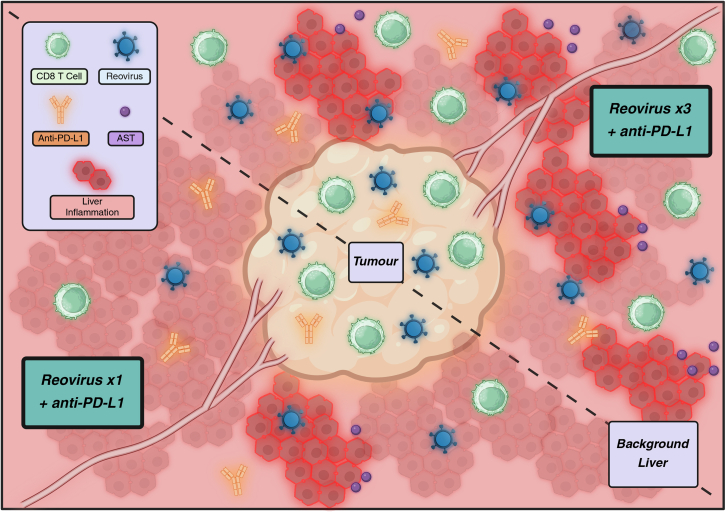
Diagram summarizing the effects of single or repeated reovirus doses, in combination with anti-PD-L1, in both tumor and background liver AST, aspartate aminotransferase. Image created in https://BioRender.com.

For HCC patients with chronically damaged background livers, a targeted approach for delivery of OVs to treat their tumors, which avoids potential unwanted toxicity to surrounding hepatic tissue, is critical for clinical benefit. Successful therapy requires the virus to be selectively delivered to the tumor site, while simultaneously achieving anti-tumor immune activation to ensure a prolonged and efficacious immune response. We demonstrate that a single dose of reovirus is as effective, if not superior, to repeated doses in: (1) inducing immune cell activation and migration, (2) ability to target tumor tissue, and (3) preventing the growth of tumors *in vivo* in combination with anti-PD-L1 therapy, thereby prolonging survival. Additionally, and potentially more importantly, a single dose reduces the impact on compromised background liver tissue, which is crucial for the effective and safe treatment of HCC patients. This work indicates the need for further virus-specific optimization of OV therapy, crucially as a single dose, in combination with checkpoint inhibitors, for immunotherapy in HCC patients. A summary diagram of our work is shown in Figure 1.

## Declaration of interests

A.S. has received research grants from Oncolytics Biotech Inc., Calgary, who provided clinical-grade pelareorep and reovirus.
